# METTL18 is Required for Developmental, Metabolic, and Endocrine Regulation of Mammalian Growth

**DOI:** 10.1096/fj.202600392R

**Published:** 2026-08-27

**Authors:** Fumiya Kasai, Jun‐Dal Kim, Koichiro Kako, Naoto Muromachi, Takeru Saito, Yao Yuan, Hayase Mizukami, Nana Kishikawa, Kanako Nakamura, Kaori Motomura, Hiroaki Daitoku, Yuuki Imai, Sho Sekiguchi, Kazuhiko Horiguchi, Satoshi Narumi, Masanobu Yamada, Akiyoshi Fukamizu

**Affiliations:** ^1^ Life Science Center for Survival Dynamics, Tsukuba Advanced Research Alliance (TARA) University of Tsukuba Ibaraki Japan; ^2^ Division of Complex Bioscience Research, Department of Research and Development Institute of National Medicine, University of Toyama Toyama Japan; ^3^ AMED‐CREST, Japan Agency for Medical Research and Development Tokyo Japan; ^4^ Institute of Life and Environmental Sciences University of Tsukuba Ibaraki Japan; ^5^ Master's Program in Agro‐Bioresource Science and Technology University of Tsukuba Ibaraki Japan; ^6^ Division of Integrative Pathophysiology Proteo‐Science Center, PIAS, Ehime University Ehime Japan; ^7^ Department of Pathophysiology Ehime University, Graduate School of Medicine Ehime Japan; ^8^ Division of Endocrinology and Metabolism, Department of Internal Medicine Gunma University Graduate School of Medicine Gunma Japan; ^9^ Department of Pediatrics Keio University School of Medicine Tokyo Japan

**Keywords:** endocrine homeostasis, histidine methylation, mammalian growth, METTL18, systemic metabolic regulation

## Abstract

Histidine methylation is an evolutionarily conserved but poorly understood post‐translational modification. METTL18 is known to catalyze histidine *N*τ‐methylation in vitro, yet its physiological functions in mammals remain unclear. Here we show that mouse METTL18 possesses conserved histidine *N*τ‐methyltransferase activity and that its genetic deletion results in widespread effects on developmental, metabolic, and endocrine function. *Mettl18* knockout (KO) embryos exhibit impaired growth with disruption of the transferrin–transferrin receptor (Trf–Tfrc) axis, suggesting insufficient fetal iron handling. After birth, *Mettl18* KO mice remain underweight and show reduced liver and white adipose tissue mass. Moreover, *Mettl18* KO mice exhibit decreased plasma and liver triglycerides, elevated adiponectin and improved glucose tolerance, indicative of a coordinated shift toward an energy‐conserving metabolic Trf–Tfrc state. Notably, METTL18 deficiency impairs IGF‐1‐mediated signaling and suppresses the TSH–T4 axis, two major endocrine pathways critical for postnatal growth. Taken together, our results indicate that METTL18 functions as a conserved histidine methyltransferase that contributes to fetal development, endocrine regulation, and systemic metabolism, thereby influencing organismal growth in mammals.

## Graphical abstract

METTL18 is essential for histidine *N*τ‐methylation of proteins, normal embryonic development, iron and lipid metabolism, and hormone signaling pathways that support postnatal growth in mice. The main points of this study are as follows: First, mouse METTL18 catalyzes histidine *N*τ‐methylation. Next, *Mettl18* KO embryos are significantly smaller than their wild‐type littermates. Moreover, Mettl18 deficiency suppresses the expression of genes associated with iron or lipid metabolism in E18.5 embryos. Interestingly, METTL18 deficiency alters systemic lipid metabolism and causes postnatal growth retardation. Finally, METTL18 is necessary for proper IGF‐1‐mediated signaling and the TSH–T4 thyroid axis during postnatal growth.

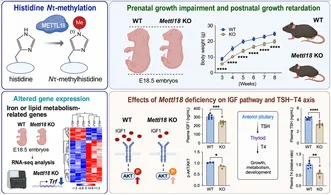

## 1 Introduction

Protein methylation is catalyzed by methyltransferase, which uses *S*‐Adenosyl‐L‐methionine (SAM) as a methyl donor, transferring a methyl group from SAM to basic amino acid residues of proteins. Although protein methylation is a widespread post‐translational modification involved in diverse aspects of cellular regulation [1, 2], histidine methylation is recognized as the third form of protein methylation, following lysine and arginine methylation, and its biochemical properties and physiological significance remain poorly understood [3, 4]. In yeast, the histidine methyltransferase Hpm1 modifies ribosomal protein Rpl3, and its loss results in impaired translation and growth [5, 6, 7, 8]. Human METTL18 was recently identified as the mammalian ortholog of Hpm1 and shown to catalyze histidine *N*τ‐methylation in vitro, suggesting that histidine methylation may represent an evolutionarily conserved regulatory mechanism [9]. While METTL18 has been biochemically characterized [10, 11], its physiological roles in mammals remain largely unknown.

The healthy development and growth of mammals rely on the coordinated control of nutrient availability, iron homeostasis, and key endocrine pathways, including the insulin‐like growth factor 1 (IGF‐1) pathway and thyroid hormone signaling, which are intrinsically linked, and nutrient and iron levels directly influence the function and regulation of these major endocrine axes [12, 13, 14, 15]. These systems act together to support fetal tissue expansion and postnatal somatic growth, yet it remains unknown whether histidine methylation participates in these fundamental processes. Given that METTL18 is expressed in multiple tissues, it may influence organismal growth by modulating cellular pathways that coordinate metabolic and endocrine homeostasis. However, no genetic studies have examined how loss of METTL18 affects fetal development, systemic metabolism, or endocrine function in vivo.

In this study, to address this question, we investigated the biological function of METTL18 in mice. We demonstrate that METTL18 possesses conserved histidine methyltransferase activity and that genetic deletion of *Mettl18* leads to broad developmental and metabolic consequences. *Mettl18* KO embryos show prenatal growth retardation associated with disruption of the transferrin–transferrin receptor (Trf–Tfrc) axis, implying insufficient fetal iron handling. Postnatally, *Mettl18* KO mice showed reduced size in multiple organs including liver, kidney, epididymal white adipose tissue (eWAT), and quadriceps muscle, and systemic metabolic alterations such as reduced plasma triglycerides (TG) and cholesterol, elevated adiponectin, and improved glucose tolerance.

Our findings uncover the first in vivo evidence that histidine methyltransferase plays an important role in mammalian physiology. By linking METTL18 to fetal growth associated with iron metabolism and systemic energy balance, this study provides a basis for further understanding the biological significance of histidine methylation.

## 2 Materials and Methods

### 2.1 cDNA Cloning and Expression of Recombinant Proteins

The human METTL18 (hMETTL18) and mouse METTL18 (mMETTL18) expression constructs were generated by PCR amplification, followed by insertion into the pGEX‐6P‐2 vector. The point mutation construct (hMETTL18 G195A and mMETTL18 G185A) was generated using PCR‐based mutagenesis. N‐terminal GST fusion proteins were expressed in BL21‐CodonPlus (DE3)‐RIL (Agilent Technologies). Briefly, they were expressed at 22°C for 4 h in the presence of 0.5 mM IPTG. Recombinant GST‐fusion proteins were purified using Glutathione Sepharose 4B (Cytiva).

### 2.2 Reagents

The primary antibodies were as follows: anti‐Transferrin (1:1000, Santa Cruz Biotechnology, #sc‐373785; RRID:AB_10918440), anti‐GAPDH (1:1000, Cell Signaling Technology, #5174; RRID:AB_10622025), anti‐Akt (1:1000, Cell Signaling Technology, #9272; RRID:AB_329827), anti‐Phospho‐Akt (Ser473) (1:1000, Cell Signaling Technology, #4060; RRID:AB_2315049), anti‐TSHβ (1:1000, Santa Cruz Biotechnology, #sc‐365801; RRID:AB_10844974), anti‐TRHR (1:1000, Sigma Aldrich, #AV‐13104; RRID:AB_1857999). Western blots were visualized by chemiluminescence using Clarity Western ECL substrate (Bio‐Rad) or ImmunoStar LD (Wako).

### 2.3 Animals

To generate the *Mettl18* deficient mice, the CRISPR‐Cas9 system was used. The 20‐mer oligonucleotides (forward: 5′‐AACGGTGTGCATAGAAAAAG‐3′ and reverse: 5′‐TAGAGCATTTGCTAGCGTGC‐3′) located upstream of the PAM site were selected as target sequences for the design of the sgRNA using CRISPRdirect [16]. The designated sgRNAs were co‐expressed with the Cas9 protein using the pX330 vector, then sgRNAs and Cas9 protein were microinjected into fertilized C57BL/6J zygotes. To validate the deletion of the *Mettl18* gene, DNA sequencing was conducted using a 3130xl Genetic Analyzer (Applied Biosystems). To minimize potential off‐target effects and passenger mutations, *Mettl18* deficient mice were backcrossed to C57BL/6J mice for over three generations prior to initiating phenotypic analysis. Genotyping was performed by amplifying the genomic DNA using PCR. The following primer sets were used: *Mettl18* forward: 5′‐GGGCTGAACTGAAAAAGGATC‐3′ and *Mettl18* reverse: 5′‐CAAGGTCCCAGGGTCTCATA‐3′.

All animal experiments were performed in a humane manner with the approval of the Institutional Animal Experiment Committee of the University of Tsukuba. Experiments were done in accordance with the Regulation of Animal Experiments of the University of Tsukuba and the Fundamental Guidelines for Proper Conduct of Animal Experiments and Related Activities in Academic Research Institutions under the jurisdiction of the Ministry of Education, Culture, Sports, Science, and Technology of Japan. All mice used were C57BL/6J background and housed under the following conditions: temperature 22°C, humidity 40%–60%, and light/dark cycle every 12 h.

### 2.4 Sampling Materials

Mouse tissues and plasma samples were collected under anesthesia with isoflurane and immediately frozen in liquid nitrogen. The frozen tissues were crushed into powder using a Multi‐Beads Shocker (Yasui Kikai), maintaining temperatures below freezing to prevent melting.

### 2.5 Grip Strength Analysis

Grip strength was measured using a dynamometer (Melquest). In brief, the mice were allowed to grab a grid connected to the dynamometer, and their tails were gently pulled backwards until they released its limbs. The maximum tension was measured three times per mouse, and the average value was calculated.

### 2.6 Histological Analyses

The liver and white adipose tissue were fixed in 4% paraformaldehyde at 4°C for 48 h, then tissues were embedded in paraffin and used for hematoxylin and eosin (H&E) staining. For Oil red O staining, the liver was immersed in 20% sucrose at 4°C for 24 h, and then embedded in OCT compound. H&E and Oil red O staining were performed using standard procedures. Images were acquired using BX53 and SZ61 microscope with DP21 digital camera (Olympus) or BZ‐X810 (Keyence). Adipocyte size analysis was performed using ImageJ 1.52 K software (National Institutes of Health; RRID:SCR_003070).

### 2.7 Metabolic and Endocrine‐Related Factor Analyses

Fetal body weight was measured by extracting the fetus via cesarean section of the mother. Postnatal body weight was measured weekly after weaning. Daily food and water intake were measured using metabolic cages. To quantify the plasma GH, mice were fasted for 16 h. For the GTT, mice were fasted for 16 h, then injected with 1 g/kg glucose intraperitoneally. Blood glucose levels were measured at specified time points using One Touch Ultra (LifeScan). Metabolic parameters and endocrine‐related factors were measured using the following kits: LabAssay Triglyceride (#632‐50991, Wako), Non‐esterified Fatty Acid (NEFA) C‐test (#279‐75401, Wako), LabAssay Cholesterol (#635‐50981, Wako), Mouse/Rat Leptin ELISA Kit (#MOB00B, R&D Systems), Mouse Adiponectin ELISA kit (#ab108785, Abcam), Mouse Insulin ELISA Kit (#10‐1247‐01, Mercodia), Rat/Mouse Growth Hormone ELISA Kit (#EZRMGH‐45K, Merck Millipore), Mouse & Rat IGF‐1/IGF‐1 ELISA Kit (#MG100, R&D Systems), Mouse Thyroid Stimulating Hormone (TSH) ELISA Kit (#KT‐29922, Kamiya Biomedical Company).

### 2.8 Liver TG, NEFA and Cholesterol Contents

Approximately 100 mg of liver sample was homogenized in distilled water, then 1:2 v/v methanol/chloroform solution was added. After centrifugation, the lower phase was collected and dried using a centrifugal concentrator (EYELA). The samples were resuspended in 10% Triton X‐100 in isopropanol, and the TG, NEFA, and cholesterol contents were determined using the LabAssay Triglyceride, NEFA C‐test, and LabAssay Cholesterol.

### 2.9 RNA Isolation and Quantitative Real‐Time PCR Analysis

Total RNA was extracted from tissues using ISOGEN II (Nippon Gene) and then reverse transcribed into cDNA using ReverTra Ace (TOYOBO). Real‐time quantitative PCR was performed using the cDNA template, and the relative mRNA expression levels were calculated based on the ΔΔCt method. The primers used in this study are seen in Table S1.

### 2.10 RNA‐Seq Analysis

Whole embryos (at embryonic day 18.5, male) were frozen in liquid nitrogen and were crushed into powder using a Multi‐Beads Shocker (Yasui Kikai). Then, total RNA was extracted using ISOGEN II. Approximately 500 ng of RNA was ribosomal RNA‐depleted using NEBNext rRNA Depletion Kit (New England Biolabs) and converted to Illumina sequencing library using NEBNext Ultra Directional RNA Library Prep Kit (New England Biolabs). RNA libraries were prepared for sequencing using standard Illumina protocols. RNA‐seq datasets were deposited in the NCBI Gene Expression Omnibus (GEO) database (www.ncbi.nlm.nih.gov/geo; accession number is GSE314501). Reads were mapped to the mouse reference genome (GRCm38/mm10) and quantified using the CLC Genomics Workbench (version 12.0, QIAGEN; RRID:SCR_000354). The read counts were normalized by calculating the number of reads per kilobase per million reads for each transcript in individual samples using CLC Genomics Workbench software (version 12.0, QIAGEN; RRID:SCR_000354). The differentially expressed genes (DEGs) were identified using fold‐change −2 to +2 filtering analysis with an FDR of *p* < 0.05.

### 2.11 In Vitro SAM Binding Assay and Automethylation Assay

In vitro SAM binding reaction was performed for autoradiography. For the former, purified GST‐hMETTL18 WT or G195A mutant, GST‐mMETTL18 WT or G185A mutant were incubated with [^3^H] SAM (*S*‐adenosyl‐L‐[*methyl*‐^3^H] methionine) (PerkinElmer) at 4°C for 3 h in PBS. The reaction products were fractionated by SDS‐PAGE. Gels stained with Coomassie Brilliant Blue staining solution were incubated for 30 min with Amplify Fluorographic Reagent (Perkin Elmer), dried, and exposed to Hyperfilm ECL (GE Healthcare) at −80°C.

In vitro automethylation reaction was performed for liquid chromatography–tandem mass spectrometry (LC–MS/MS) analysis. GST‐hMETTL18 WT or G195A mutant, GST‐mMETTL18 WT or G185A mutant were incubated at 30°C for 2 h, in the presence of cold SAM in PBS. The reactions were separated by SDS‐PAGE, and the gels were negatively stained with EzStain Reverse (ATTO). The gel bands derived from GST‐METTL18 were excised and crushed into a gel slurry, and then the proteins were purified using ATTOPREP MF (ATTO). The resulting proteins were subjected to acid hydrolysis followed by LC–MS/MS analysis.

### 2.12 Extraction of Thyroid Hormones From Mouse Plasma

A 100 μL of plasma was spiked with 2 μL of 100 μM Fmoc‐Tyr(tBu)‐OH (a tyrosine derivative, Peptide Research Institute) as an internal standard. IS‐spiked plasma was mixed vigorously with 100 μL of 5% NH_4_OH/acetonitrile, then centrifuged at 17 800 *g* at 4°C for 5 min. Recovered 170 μL of supernatant was mixed with 1.5 mL of 12.5% NH_4_OH, then vortexed vigorously, and the sample was loaded onto an Oasis HLB (Waters). After neutralizing this column with 1 mL of acetic acid and washing with 3 mL of 40% methanol to remove hydrophilic impurities, the crude thyroid hormone fractions were eluted with 2 mL of methanol. The eluent was then concentrated and dried up by centrifugation using a centrifugal concentrator. The sample was stored at −20°C until analysis by LC–MS/MS.

### 2.13 Extraction and Semi‐Purification of TRH From Mouse Whole Brain

Dissected whole brains from WT and *Mettl18* KO mice were immediately frozen in liquid nitrogen. After crushing the frozen brain tissue using the Multi‐Beads Shocker at 2000 shakings per minute for 10 s kept chill, half of the brain powder was suspended in 1 mL of distilled water on ice. The crude extracts were spiked with 5 μL of 10 μM saralasin (NH_2_‐Sar‐Arg‐Val‐Tyr‐Val‐His‐Pro‐Ala‐COOH, Peptide Research Institute) as an internal standard and then sonicated for 5 min using a BIORUPTOR UCD‐250 (Cosmo Bio). After centrifugation of the extract at 15,000 *g* at 4°C for 15 min, 600 μL of supernatant was recovered, then mixed with an equal volume of boiling 2 M acetic acid‐40 mM hydrochloric acid followed by further boiling at 99°C for 10 min to denature and inactivate proteases and other enzymes and proteins. The extract was mixed with 600 μL of 1 M acetic acid and 1.8 mL of diethyl ether vigorously, then centrifuged at 715 *g* for 10 min at room temperature. The recovered 1.8 mL of the aqueous layer was mixed with an equal volume of CHCl_3_:methanol (2:1), then centrifuged at 715 *g* for 10 min at room temperature and collected 1.8 mL of the upper layer. The above aqueous layer was applied to Phree Phospholipid Removal (Phenomenex), conditioned with 1% formic acid/acetonitrile, to remove phospholipids. The eluate was loaded on a 0.1% trifluoroacetate (TFA)‐conditioned Sep‐Pak Plus C_18_ Cartridge (Waters) and eluted using 80% acetonitrile/0.1% TFA to remove hydrophilic contaminants, then the peptide fraction was concentrated and dried by centrifugation using a CENTRIFUGAL VAPORIZER CVE‐200D (Tokyo Rika). The sample was stored at −20°C until analysis by LC–MS/MS.

### 2.14 LC–MS/MS Analyses

All LC–MS/MS analyses were performed using a Nexera X2‐LCMS‐8050 (Shimadzu Corporation). Detection was performed in positive ion mode for all samples. The MS/MS section was optimized as follows: heating block temperature: 400°C, desolvation line temperature: 250°C, ion source temperature: 300°C, ionization voltage: 4.0 kV, ionization current: 0.1 μA, heating gas (air) flow rate: 10 L/min, nebulizer gas (N_2_) flow rate: 3 L/min, drying gas (N_2_) flow rate: 10 L/min, collision‐induced dissociation (CID) Ar gas pressure: 270 kPa. Optimal conditions for each measured ion were established via precursor ion scans and product ion scans in multiple reaction monitoring (MRM). Mass spectrometry performed CID on the precursor ion, selectively detecting the resulting product ions. Ion intensity was plotted over time to obtain a MS chromatogram. Quantification of each analyte was derived by creating calibration curves within the range where a linear relationship existed between peak areas of known concentration standards and their respective concentrations. All instrument control and data processing were performed using LabSolutions ver. 5.60 software (Shimadzu Corporation).

For the analyses of thyroid hormones, dried sample was dissolved in 25 μL of distilled water, and 10 μL of sample was injected onto Inertsil ODS‐HL (3 μm, 2.1 × 150 mm, GL Sciences) column kept at 30°C. Mobile phase A contained 1% formic acid, while mobile phase B contained 1% formic acid‐99% acetonitrile. The column was maintained at 30% B for 5 min after sample injection, followed by a gradient elution to 25% B over 20 min. This was immediately followed by a 10‐min hold at 100% B, then a 5‐min column flush at 30% B. Quantitative results were normalized using Fmoc‐Tyr(tBu)‐OH as an internal standard. The MRM transitions for each analyte using standards were thyroxine (T4, Sigma‐Aldrich): *m/z* 777.5 > 731.7, triiodothyronine (T3, Sigma‐Aldrich): *m/z* 651.7 > 605.8 and Fmoc‐Tyr(tBu)‐OH: *m/z* 460.0 > 179.2.

For the analyses of TRH, the dried peptide fraction was suspended in 25 μL of distilled water, and 10 μL of the suspension was injected onto Inertsil ODS‐HL as described above. Mobile phase A contained 1% formic acid, while mobile phase B contained 1% formic acid in 99% acetonitrile. The column was maintained at 0% B for 5 min after sample injection, followed by a gradient elution to 50% B over 25 min, then immediately followed by a 10‐min hold at 100% B and a final 10‐min column re‐equilibration at 0% B. The obtained results were corrected for total protein content and saralasin. The MRM transitions used for each analyte with each standard were: TRH (Peptide Research Institute): *m/z* 363.0 > 249.2 and saralasin: *m/z* 456.8 > 187.1 ([M + 2H]^2+^). This TRH detection method was validated using prepro‐TRH knockout mice: a clear TRH peak was detected in wild‐type brain extracts but was absent in knockout samples (Figure S1).

Quantitative analyses of *N*τ‐methylhistidine were carried out according to a previously reported method [17, 18]. Briefly, samples were acid‐hydrolyzed with 6 M of hydrochloric acid at 110°C for 24 h, dissolved in 50 μL of water, and 10 μL of sample injected onto a SeQuant ZIC‐HILIC column (2.1 × 150 mm, 3.5 μm; Merck KGaA) maintained at 40°C. Chromatography conditions were as previously reported [17, 18]. *N*‐propyl‐l‐arginine (*N*‐PLA, Sigma‐Aldrich) was added at 25 ng immediately prior to acid hydrolysis as an internal standard. The MRM transitions for each analyte using each standard were determined as *m/z* 217.2 > 70.2 for *N*‐PLA, *m/z* 156.1 > 110.1 for histidine (Sigma‐Aldrich), *m/z* 170.1 > 124.1 for *N*τ‐methylhistidine (Sigma‐Aldrich). All solvents used in this study were analytical reagent grade.

### 2.15 Statistical Analysis

All statistical analyses were performed using GraphPad Prism 9 (GraphPad Software; RRID:SCR_002798). The data were analyzed with unpaired Student's *t*‐test, one‐way ANOVA or two‐way repeated measures of ANOVA followed by the Tukey's multiple comparison test. Results with *p < 0.05, **p < 0.01, ***p < 0.001, and ****p < 0.0001 were considered statistically significant.

## 3 Results

### 3.1 METTL18 Is a Conserved Histidine *N*τ‐Methyltransferase in Mice

**FIGURE 1:**
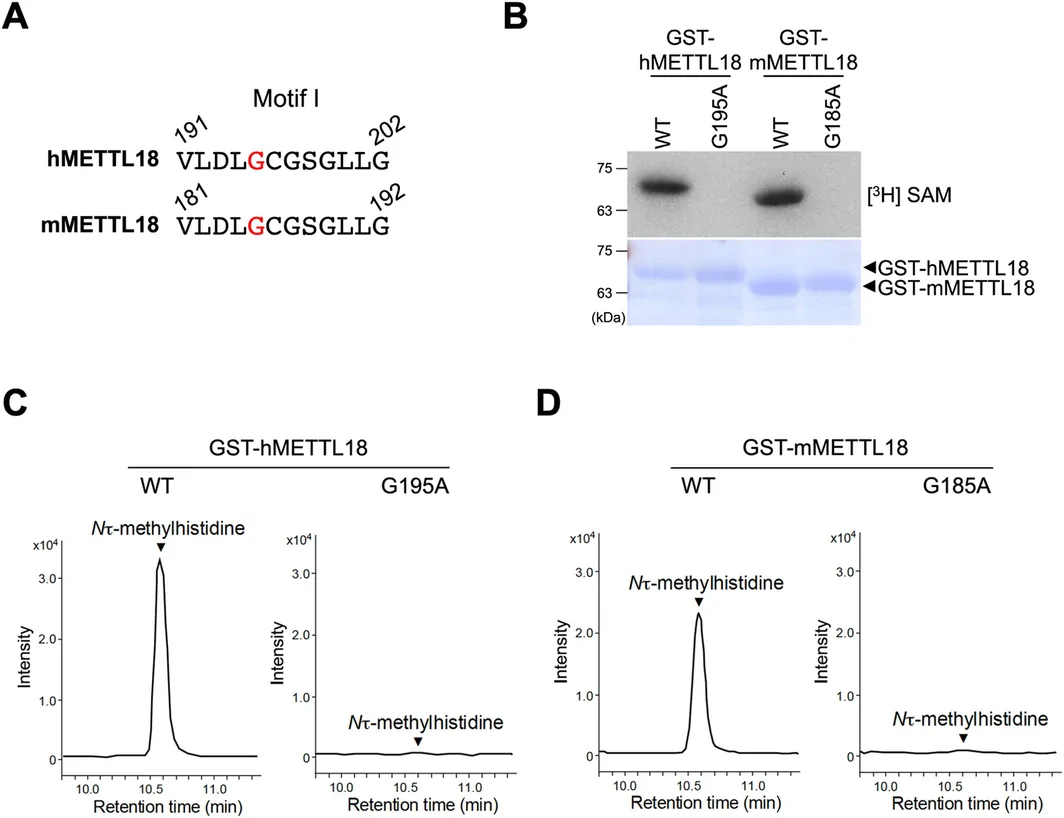
METTL18 harbors conserved catalytic motifs and catalyzes His *N*τ‐methylation in vitro. (A) Sequence alignment of SAM binding motif (Motif I) for human and mouse METTL18 proteins, including the invariant glycine residues mutated in this study. (B) In vitro SAM binding assays of GST‐tagged human and mouse METTL18 wild‐type (WT) and catalytic mutants (G195A for human, G185A for mouse) using [^3^H]‐SAM as methyl donor. (C, D) LC–MS/MS detection of automethylated histidine products generated by human (C) or mouse METTL18 (D).

Previous studies reported that human METTL18 and its yeast ortholog (Hpm1) are histidine *N*τ‐methyltransferases and they have automethylation activity [5, 9, 10]. Considering this, we first examined whether the mouse METTL18 also has protein histidine methyltransferase activity in vitro. Human and mouse METTL18 have highly similar amino acid sequences, and the motifs that are critical for SAM binding (Motif I) and substrate recognition (Motif II) are completely identical (Figure 1A and Figure S2A). To test whether mouse METTL18 catalyzes protein methylation, we performed an in vitro SAM binding assay by using recombinant human and mouse METTL18 in the presence of [^3^H] SAM. As illustrated in Figure 1B, we detected radiolabeled SAM signal of wild‐type human and mouse METTL18, whereas no signals were detected in inactive mutants of METTL18 by one amino acid substitution in SAM binding motif (human; G195A, mouse; G185A). Next, to identify the type of methyl amino acids produced by automethylation of METTL18, we analyzed the acid hydrolysate of post‐reaction human and mouse METTL18 using LC–MS/MS. As shown in Figure 1C,D, strong peaks of *N*τ‐methylhistidine only in the wild‐type human and mouse METTL18 samples were observed. In this way, these data support that mouse METTL18 possesses histidine *N*τ‐methylation activity.

### 3.2 METTL18 Deficiency Causes Prenatal Growth Impairment

**FIGURE 2:**
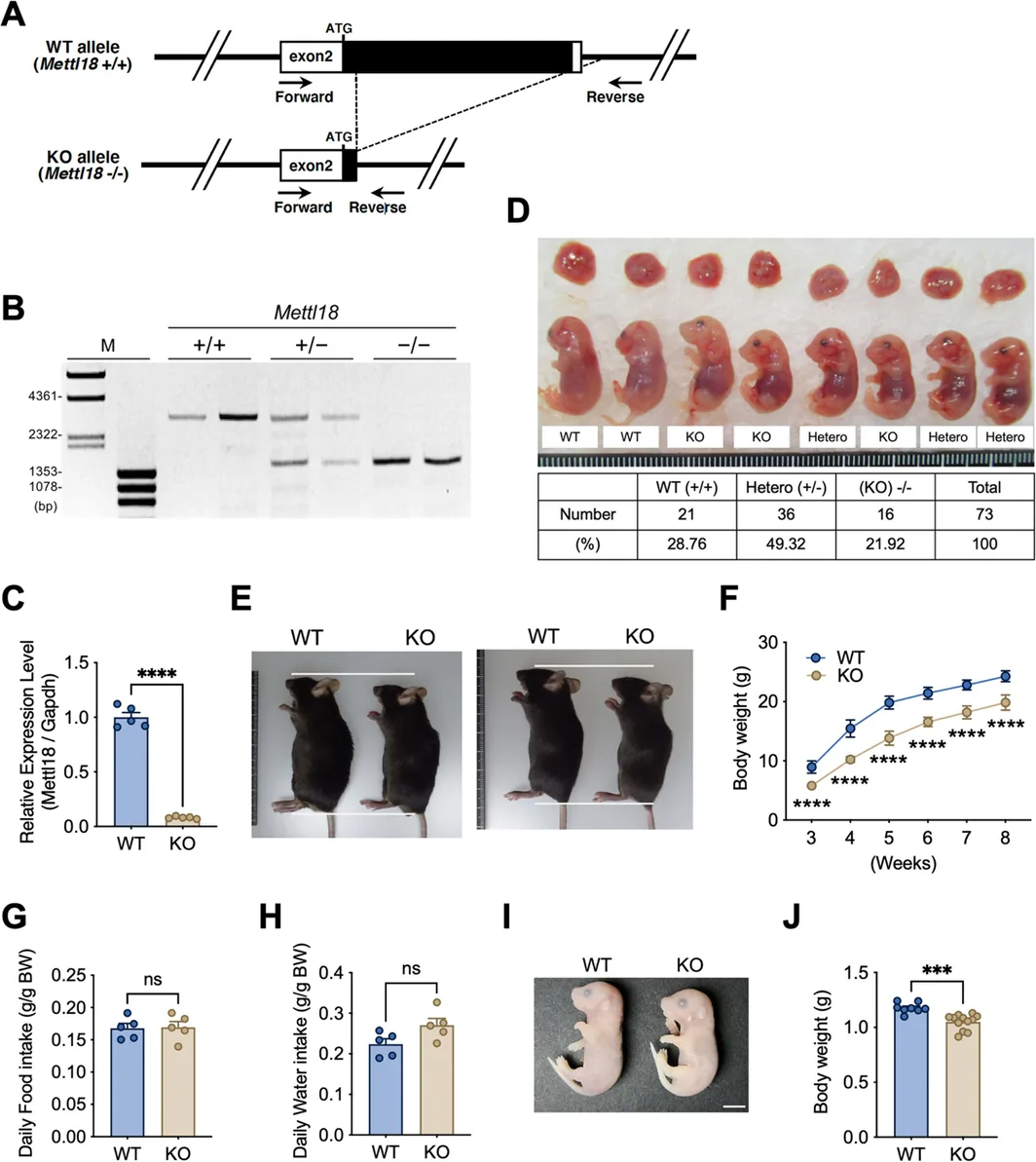
*Mettl18* is required for prenatal and postnatal development of mammals. (A) Schematic of the CRISPR/Cas9‐generated *Mettl18* KO allele showing deletion within exon 2. Arrows indicate positions of PCR primers used for genotyping. (B) Representative genotyping PCR confirming wild‐type *Mettl18* (+/+, WT), heterozygous (+/−, Hetero), and homozygous (−/−, KO) alleles. (C) Quantitative PCR showing complete loss of *Mettl18* expression in KO embryos. Data are presented as mean ± standard errors of the mean (SEM) for *n* = 5 per group. (D) E18.5 littermates. Representative WT, *Mettl18* Hetero, and *Mettl18* KO embryos. The genotypic distribution of heterozygous offspring. (E) Male (left) and female (right) views of representative 8‐week‐old WT and *Mettl18* KO mice. (F) Growth curve of *Mettl18* WT and KO littermates from 3 to 8 weeks. Data are presented as mean ± SEM for *n* = 5 per group. (G, H) Daily food (G) and water intake (H) normalized to body weight. No significant (ns) changes were observed between genotypes (*n* = 5). (I, J) Representative images of WT and *Mettl18* KO embryos at E18.5 (I) and quantitative comparison of body weight (J). Scale bars, 1 cm. Data are presented as mean ± SEM for *n* = 8–12 per group. ***p < 0.001 and ****p < 0.0001 versus WT control. The effect size of Cohen's *d* = 2.0493. Statistical analyses were performed using unpaired two‐tailed Student's *t*‐tests or two‐way repeated measures of ANOVA with Tukey's multiple comparison test.

To elucidate the in vivo function of METTL18, we first examined the expression pattern of METTL18 mRNA in mouse tissues. In male and female C57BL/6J mice, the METTL18 mRNA expression was broadly detected across tissues (Figure S2B,C). Next, to examine the function of METTL18 at organismal levels, we generated the *Mettl18* knockout (KO) mice using the CRISPR‐Cas9 system (Figure 2A,B). *Mettl18* mRNA levels were significantly decreased in the whole tissue of *Mettl18* KO mice embryos compared with control mice (Figure 2C). As shown in Figure 2D, *Mettl18*‐deficient mice exhibit typical Mendelian inheritance, with no evidence of prenatal lethality. *Mettl18* KO mice were normally viable, but they were underweight (Figure S3A–C), and the growth retardation continued after birth despite no change in food and water intake (Figure 2E–H). Importantly, at embryonic day 19 (E19), *Mettl18* KO embryos were already significantly smaller than their wild‐type (WT) littermates (Figure 2I,J), suggesting that the growth defect arises, at least in part, during fetal development.

### 3.3 Mettl18 Deficiency Suppresses the Expression of Iron or Lipid Metabolism‐Associated Genes in the Embryos

**FIGURE 3:**
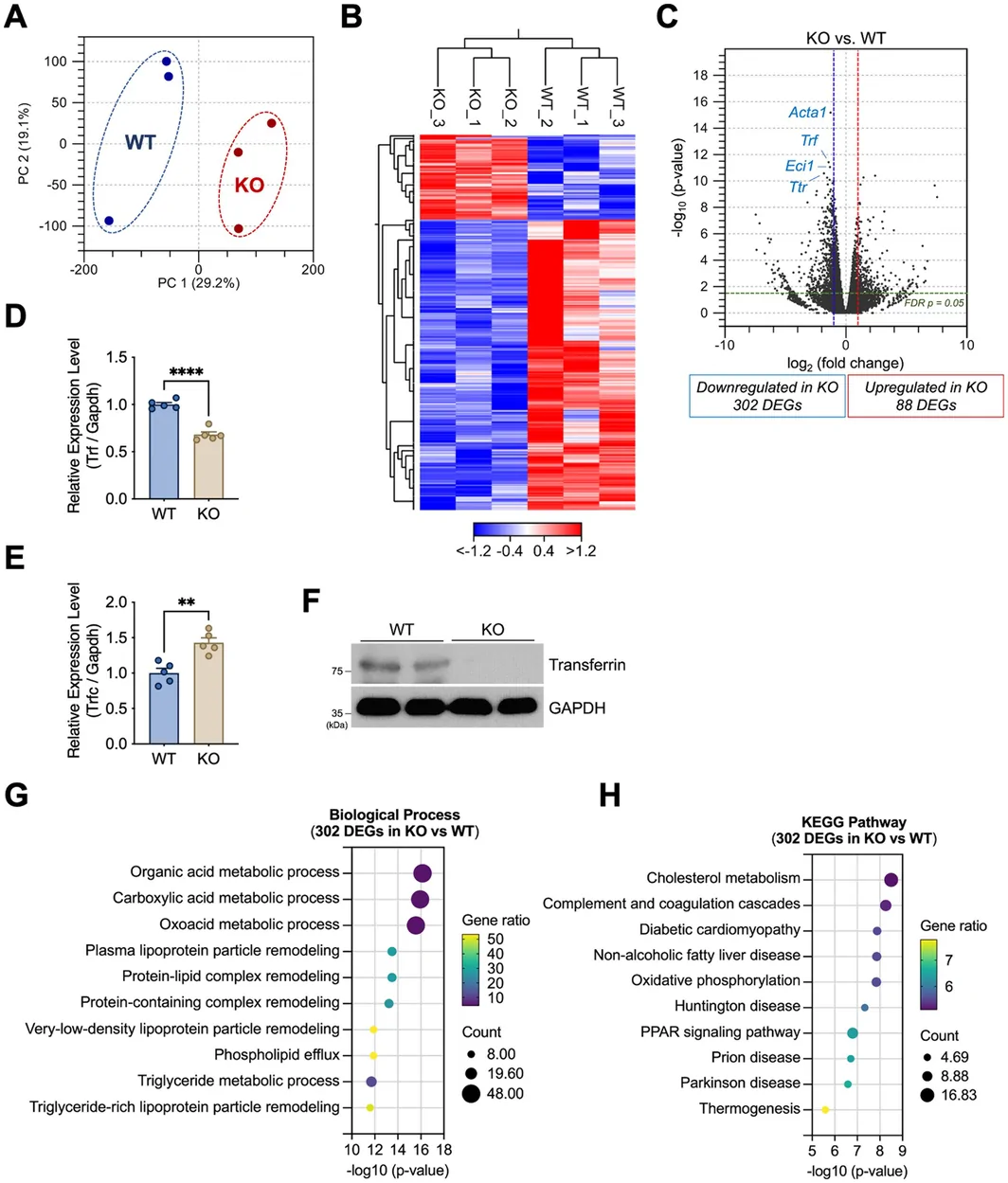
*Mettl18* deficiency causes changes of iron and lipid metabolism‐related gene in embryos. (A) Principal component analysis (PCA) of the RNA‐seq data. Principal component 1 (PC1) (*x*‐axis) and PC2 (*y*‐axis) account for 29.2% and 19.1% of the variation between *Mettl18* WT and KO groups. Each dot denotes a single biological replicate, and the dashed circles represent three replicates for each sample. Blue dots; WT group and red dots; *Mettl18* KO group. (B) Hierarchical clustering of the expression profiles between two groups. Individual samples are provided in columns and genes in rows. Heatmaps represent the relative expression (red; high, white; intermediate, and blue; low expression). (C) Volcano plots represent the differentially expressed genes (DEGs) between *Mettl18* WT and KO groups. Dotted vertical lines, log_2_ FC ≥ 2 or ≤ −2; dotted horizontal line, the significance cut‐off (false discovery rate: *P* = 0.05). (D, E) qPCR validation demonstrating reduced *Trf* (transferrin) (D) and induced *Tfrc* (transferrin receptor) (E) expression in KO embryos. Data are presented as mean ± SEM for *n* = 5 per group. **p < 0.01, ****p < 0.0001 versus WT control. Statistical analyses were performed using unpaired two‐tailed Student's *t*‐tests. (F) Immunoblotting confirms decreased transferrin protein levels in *Mettl18* KO embryos (*n* = 2). (G, H) Functional enrichment analysis of 302 downregulated DEGs in the *Mettl18* KO group versus WT group, the negative log10 of the *p*‐value. The top 10 enriched gene ontology (GO) terms associated with biological process (G) and Kyoto Encyclopedia of Genes and Genomes (KEGG) pathway (https://www.kegg.jp/kegg/kegg1.html) analysis (H).

To gain insight into how *Mettl18* deficiency alters embryonic transcripts and the growth, we conducted RNA sequencing in E18.5 embryos of WT and *Mettl18* KO groups. To assess the reproducibility of biological replicates between two groups, we performed clustering using principal component analysis (PCA) and hierarchical clustering analysis (Figure 3A,B). Both analyses revealed distinct separation between the two groups, indicating robust differences in gene expression profiles. Volcano plot analysis further revealed 390 differentially expressed genes (DEGs) between the WT and *Mettl18* KO groups, with 88 upregulated and 302 downregulated in the *Mettl18* KO group, and *Acta1*, *Trf*, *Ecl1*, and *Ttr* were significantly downregulated in *Mettl18* KO embryos (Figure 3C and Table S2). It has been reported that dysfunction of iron homeostasis and metabolism by deficiency of transferrin receptor 1 (*Tfrc*) leads to growth arrest [19]. To validate these findings in *Mettl18* KO embryos, we assessed the expression of *Trf* and *Tfrc*, key mediators of systemic iron transport and cellular uptake. Notably, quantitative PCR confirmed a significant reduction of *Trf* mRNA levels, while *Tfr*c mRNA levels were concomitantly upregulated in *Mettl18* KO embryos compared with WT controls (Figure 3D,E). Moreover, Western blotting revealed dramatically decreased transferrin protein levels in *Mettl18* KO embryos (Figure 3F), suggesting that *Mettl18* is involved in the regulation of the transferrin–transferrin receptor axis, which may affect iron transport during fetal development.

Consistently, to characterize the functional features of the downregulated 302 DEGs, we conducted gene ontology (GO) annotation and Kyoto Encyclopedia of Genes and Genomes (KEGG) pathway enrichment analyses. The biological processes enriched in the 302 DEGs included “Phospholipid efflux”, “Triglyceride metabolic process”, and “Triglyceride‐rich lipoprotein particle remodeling” (Figure 3G). KEGG pathway analysis further identified terms such as “Cholesterol metabolism”, “Oxidative phosphorylation”, and “Thermogenesis” as significantly enriched (Figure 3H). These data suggest that METTL18 influences the expression of genes involved in lipid metabolism during prenatal growth.

### 3.4 METTL18 Deficiency Modulates Systemic Lipid Metabolism

**FIGURE 4:**
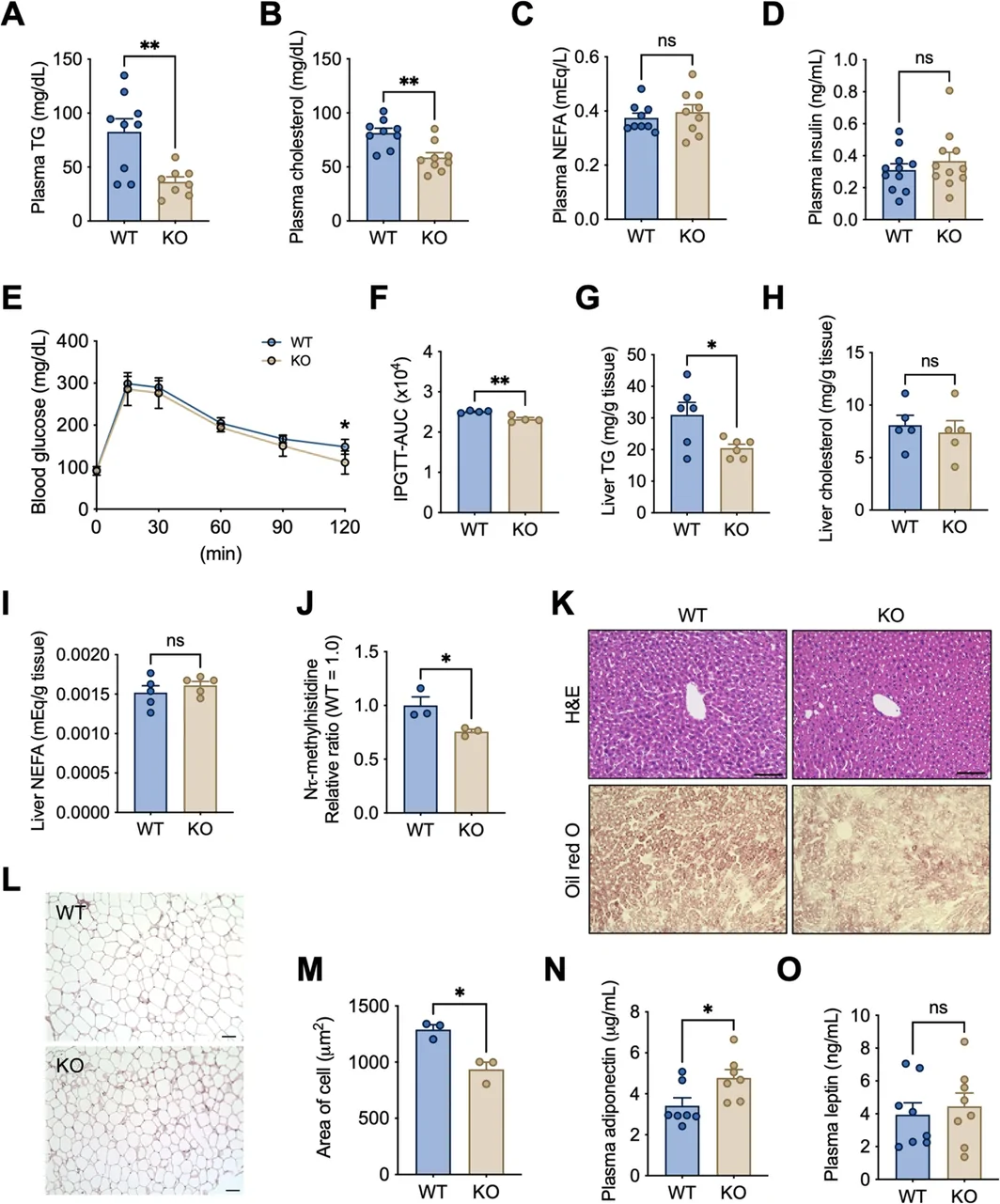
Loss of METTL18 alters lipid metabolic parameters. (A–D) Quantification of plasma triglycerides (TG) (A), cholesterol (B), NEFA (C), and insulin (D) levels under fed conditions. Data are presented as mean ± SEM for *n* = 8–11 per group. The effect size of Cohen's *d* = 1.6589 (A), 1.7097 (B), 0.3142 (C), and 0.3600 (D). (E, F) Intraperitoneal glucose tolerance test (GTT) in 16‐week‐old male mice (E), area under the curve (AUC) from the GTT (F). Data are presented as mean ± SEM for *n* = 4 per group. (G–I) Quantification of hepatic TG (G), cholesterol (H), and NEFA (I) levels under fed conditions in 8‐week‐old male mice. Data are presented as mean ± SEM for *n* = 5–6 per group. The effect size of Cohen's *d* = 3.1772 (F), 1.4627 (G), 0.3000 (H), and 0.5700 (I). (J) Quantification of liver *N*τ‐methylhistidine levels. Data are presented as mean ± SEM for *n* = 3 per group. The effect size of Cohen's *d* = 2.3958. (K) Liver histology. Representative micrographs showing H&E (upper images) and Oil red O (lower images) staining on liver sections from 8‐week‐old male WT and *Mettl18* KO mice. (L, M) Representative images of H&E staining of eWAT sections showing adipocyte lipid depots (L) and morphometric analysis of average adipocyte size (M). Data are presented as mean ± SEM for *n* = 3 per group. (N, O) Quantification of plasma adiponectin (N) and leptin (O) levels under fed conditions in 8‐week‐old male mice. Data are presented as mean ± SEM for *n* = 7–8 per group. *p < 0.05, **p < 0.01, and ns (not significant) versus WT control. Statistical analyses were performed using unpaired two‐tailed Student's *t*‐tests. The effect size of Cohen's *d* = 1.3039 (N) and 0.2303 (O). Scale bars, 50 μm.

To explore potential factors underlying the low body weight in *Mettl18* KO mice, we measured their tissue weight and revealed hypoplastic liver, kidney, eWAT, and quadriceps muscle at two months of age. *Mettl18* KO mice exhibit lower weights of liver, kidney, and eWAT than WT mice, indicating that METTL18 is involved in energy metabolism (Figure S3D). Therefore, to investigate effects of METTL18 on systemic lipid and glucose metabolism, we measured blood and liver parameters. As shown in Figure 4A–D, the *Mettl18* KO mice exhibited significantly decreased levels of plasma TG and cholesterol, but no changes in NEFA and insulin levels compared to controls. Furthermore, *Mettl18* KO mice showed no changes in blood glucose levels compared to controls, but improved glucose tolerance (Figure S4A, Figure 4E,F). When compared to WT group, the hepatic TG was significantly decreased in *Mettl18* KO mice, but no total cholesterol and NEFA (Figure 4G–I). Additionally, in the livers of *Mettl18* KO mice, *N*τ‐methylhistidine levels were slightly lower than that in the WT control group (Figure 4J). The liver weight and size of *Mettl18* KO mice were lower and smaller than those of WT mice (Figure S3D,E). Besides, H&E and Oil red O staining revealed significant vacuole and lipid droplet reduction in the liver of *Mettl18* KO mice (Figure 4K).

Given the reduced eWAT mass observed in *Mettl18* KO mice (Figure S3D), we next examined adipocyte morphology. The eWAT in *Mettl18* KO mice exhibited marked hypoplasia (Figure S3E), and adipocytes displayed significantly decreased cell size (Figure 4L,M). To assess whether altered adipogenic gene expression contributes to the phenotype of the adipose tissue hypoplasia, we measured the expression levels of genes involved in fat synthesis and degradation, but there were no significant differences between *Mettl18* KO and control mice (Figure S3F). On the other hand, adipose tissue hypoplasia is known to often alter the secretion of the adipocytokines [20]. Indeed, *Mettl18* KO mice showed a significant increase of adiponectin in plasma (Figure 4N). We also measured the leptin levels, but there were no significant differences between *Mettl18* KO and control mice (Figure 4O). Collectively, these results suggest that METTL18 contributes to systemic lipid metabolic regulation.

### 3.5 METTL18 Is Required for Proper IGF‐1‐Mediated Signaling During Postnatal Growth

**FIGURE 5:**
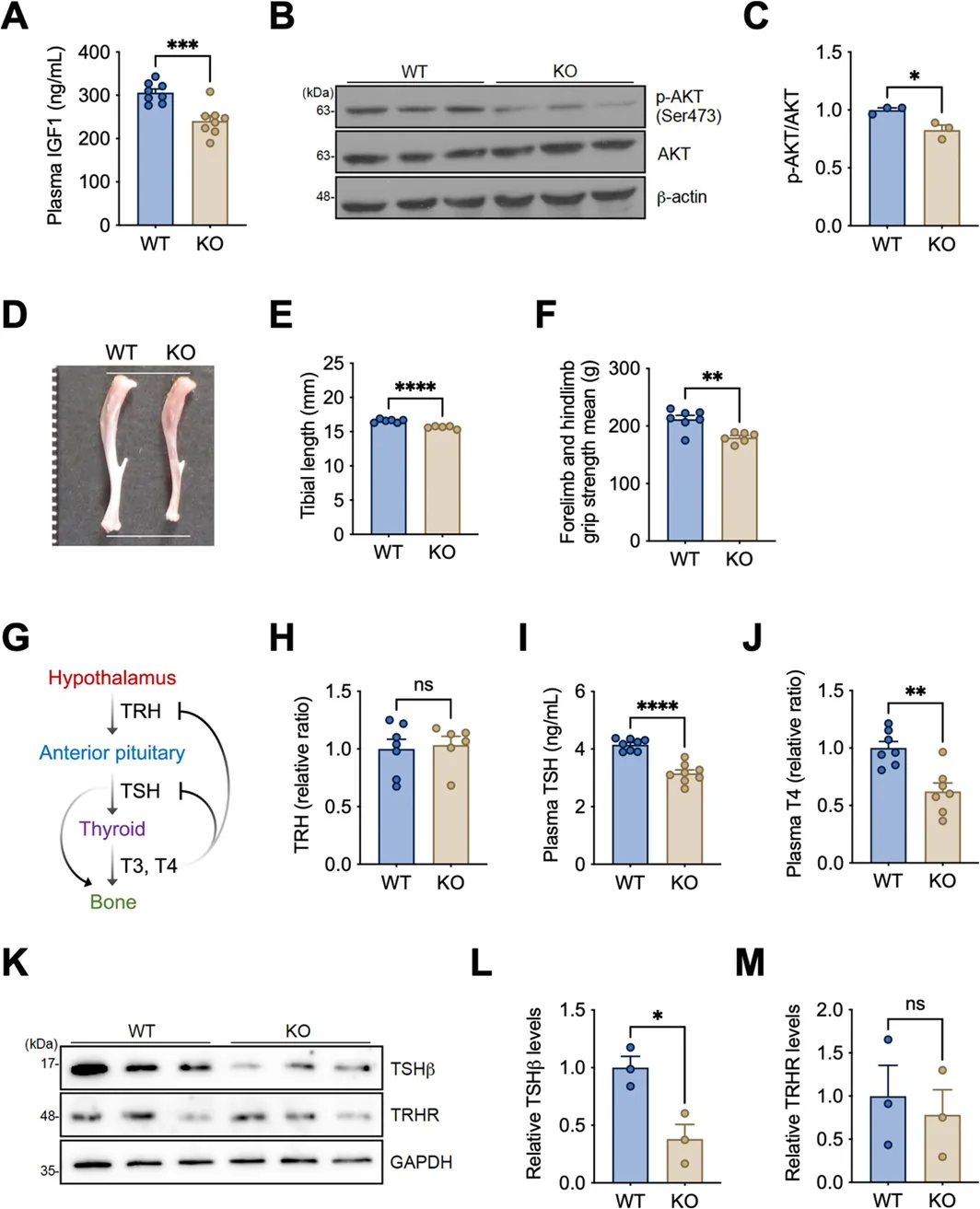
METTL18 deficiency causes a central hypothyroid‐like state with reduced TRH–TSH–T4 axis activity. (A) Quantification of plasma IGF‐1 concentrations in 8‐week‐old male WT and *Mettl18* KO mice. Data are presented as mean ± SEM for *n* = 8 per group. The effect size of Cohen's *d* = 2.2069. (B, C) Western blot analysis of hepatic AKT and phosphorylation of Ser473 in AKT (B) and quantification of p‐AKT/AKT ratio (C). Data are presented as mean ± SEM for *n* = 3 per group. The effect size of Cohen's *d* = 3.0675. (D, E) Representative photographs of hindlimbs (D) and quantitative measurement of tibial length in 8‐week‐old male mice (E). Data are presented as mean ± SEM for *n* = 5–6 per group. The effect size of Cohen's *d* = 4.1711. (F) Forelimb and hindlimb grip strength measurements in 8‐week‐old male mice. Data are presented as mean ± SEM for *n* = 6–7 per group. The effect size of Cohen's *d* = 2.1639. (G) The schematic diagram represents the hypothalamus–pituitary–thyroid (HPT) axis. (H) LC–MS/MS quantification of brain TRH content in 8‐week‐old male mice. Data are presented as mean ± SEM for *n* = 6–7 per group. ns (not significant) versus WT control. The effect size of Cohen's *d* = 0.1683. (I, J) Quantification of plasma TSH (I) and T4 (J) levels in 8‐week‐old male mice. Data are presented as mean ± SEM for *n* = 7–8 per group. The effect size of Cohen's *d* = 3.5853 (I) and 2.1596 (J). (K–M) Representative Western blotting image (K) and densitometry of pituitary TSHβ (L) and TRHR (M) proteins in 8‐week‐old male mice relative expression versus GAPDH protein. Data are presented as mean ± SEM for *n* = 3 per group. *p < 0.05, **p < 0.01, ***p < 0.001, ****p < 0.0001 and ns (not significant) versus WT control. The effect size of Cohen's *d* = 3.1696 (L) and 0.3874 (M). Statistical analyses were performed using unpaired two‐tailed Student's *t*‐tests.

As persistent postnatal growth retardation was evident in *Mettl18* KO mice, we next investigated whether endocrine factors involved in systemic growth regulation were altered. Additionally, growth hormone (GH) and insulin‐like growth factor‐1 (IGF‐1) constitute a major hormonal axis controlling somatic growth [21]. As shown in Figure S4B, plasma GH concentrations did not differ between WT and *Mettl18* KO mice under either ad libitum–fed or overnight fasting conditions. Thus, *Mettl18* deficiency does not appear to alter basal GH levels or the fasting‐induced GH response. By contrast, circulating IGF‐1 levels and its downstream AKT phosphorylation were significantly reduced in livers of *Mettl18* KO mice (Figure 5A–C), indicating impaired IGF‐1‐mediated signaling despite preserved GH levels.

### 3.6 METTL18 Deficiency Selectively Impairs the TSH–T4 Thyroid Axis

Thyroid hormones play essential roles in postnatal growth, metabolic rate, tissue maturation, and development of bone [22]. Moreover, IGF‐1 mediates the well‐known effects of GH on peripheral conversion of plasma thyroxine T4 to T3 [23]. *Mettl18* KO mice also had shortened tibial length and reduced limb grip strength (Figure 5D–F). Therefore, to assess whether thyroid hormone signaling contributes to the reduced postnatal growth of *Mettl18* KO mice, we focused on the integrity of the hypothalamic–pituitary–thyroid (HPT) axis (Figure 5G). Hypothalamic expression of thyrotropin‐releasing hormone (TRH) was comparable between WT and *Mettl18* KO mice (Figure 5H), indicating that hypothalamic input to the pituitary is preserved. In contrast, circulating TSH levels were substantially decreased in *Mettl18* KO mice (Figure 5I). Consistent with reduced pituitary output, T4 concentrations were markedly lower in *Mettl18* KO mice (Figure 5J), demonstrating reduced thyroid hormone production. At the pituitary level, immunoblot analysis revealed reduced abundance of TSHβ protein in *Mettl18* KO mice, whereas expression of TRH receptor (TRHR) was unchanged at the protein level (Figure 5K–M). These findings suggest that the decrease in circulating TSH reflects impaired TSH synthesis and/or secretion rather than defective TRH responsiveness.

## 4 Discussion

Histidine methylation is a rare and understudied post‐translational modification, and the physiological roles of the underlying enzymes remain largely unresolved [3, 4, 24]. Although METTL18 has been implicated in histidine methylation in yeast and human cells, whether and how histidine methylation contributes to mammalian physiology has remained unknown [5, 6, 9, 10, 11]. By generating and characterizing *Mettl18* KO mice, our study fills this major gap and reveals essential roles for METTL18 in fetal development, endocrine regulation, and metabolic homeostasis. Specifically, our study suggests that METTL18 serves as an integrative regulator of growth, where its deficiency leads to coordinated defects across development, metabolism, and endocrinology. During the fetal stage, loss of METTL18 downregulates Trf, disrupting the Trf–Tfrc axis and limiting the iron supply necessary for prenatal development. Postnatally, METTL18 deficiency impairs pituitary endocrine competency, suppressing the TSH–T4 axis and reducing IGF‐1 production to directly restrict somatic growth. Concurrently, altered hepatic lipid metabolism and adipose hypoplasia signal a shift toward an energy‐conserving metabolic state to adapt to this growth deficit. Although the precise molecular mechanisms linking METTL18's methyltransferase activity to these downstream transcriptional changes remain to be fully elucidated, our findings demonstrate that these distinct cellular functions act in concert to drive the complex growth retardation phenotype observed in *Mettl18* KO mice.

*Mettl18* KO embryos exhibited marked growth retardation by late gestation. Transcriptomic and biochemical analyses revealed reduced *Trf* expression and compensatory upregulation of *Tfrc*, suggesting impaired fetal iron handling. Because iron availability is essential for mitochondrial respiration [25], cellular proliferation [26], and embryonic growth [27], disruption of the Trf–Tfrc axis likely contributes to the observed fetal hypoplasia [28]. These findings indicate that even modest disturbances in transferrin‐mediated iron handling can restrict embryonic growth potential. Evidence from mouse models further supports the idea that efficient transferrin‐dependent iron homeostasis is a limiting factor for fetal growth. For example, impaired transferrin receptor recycling leads to defective cellular iron uptake and significantly reduced body size at late embryonic and neonatal stages [29], highlighting the sensitivity of fetal development to disruption of the Trf–Tfrc axis. Moreover, transferrin itself functions as a growth‐promoting factor during organogenesis, stimulating proliferation and differentiation in fetal tissues [27]. Within this framework, the altered Trf–Tfrc balance observed in *Mettl18* KO embryos is likely to compromise fetal iron availability, providing a mechanistic basis for their prenatal hypoplasia. Although the molecular link between METTL18 and iron metabolism remains unclear, the convergence of transcriptomic signatures and disrupted Trf–Tfrc balance suggests that METTL18 may act upstream of, or in parallel with, pathways involved in fetal iron handling.

Despite normal food and water intake, *Mettl18* KO mice remained smaller than WT littermates throughout life and displayed reduced adipose tissue mass, smaller adipocytes, decreased plasma TG and cholesterol, and elevated adiponectin. Together, these features suggest a metabolic shift toward an energy‐conserving, lipid‐sparing state that limits anabolic capacity. Increased adiponectin, known to enhance fatty acid oxidation and insulin sensitivity [30], likely contributes to improved glucose tolerance in *Mettl18* KO mice. These findings imply that METTL18 helps maintain adequate anabolic capacity and lipid availability during postnatal growth.

Our findings identify links between METTL18 and endocrine systems influencing postnatal growth that have not been explored. Although GH secretion was preserved under both fed and fasting conditions, circulating IGF‐1 levels and hepatic AKT phosphorylation were significantly reduced in *Mettl18* KO mice. Because IGF‐1 is the primary mediator of GH‐dependent anabolic signaling [31], impaired IGF‐1 availability and downstream signaling provide a mechanistic explanation for the reduced somatic growth of *Mettl18* KO mice, even despite normal GH levels. Importantly, the endocrine profile of *Mettl18* KO mice is consistent with central hypothyroidism arising at the level of the pituitary. While hypothalamic TRH expression remained unchanged, pituitary TSHβ protein abundance and circulating TSH and T4 were markedly reduced, indicating impaired pituitary thyrotrope output. A similar endocrine configuration has been reported in mice lacking the thyrotropin‐releasing hormone receptor (TRHR), a well‐established model of pituitary‐level central hypothyroidism [32]. TRHR‐deficient mice exhibit markedly reduced circulating T3 and T4 despite preserved hypothalamic TRH expression, indicating that impaired thyrotrope output arises from defective pituitary signaling rather than diminished hypothalamic drive. The resemblance between *Mettl18* KO and TRHR‐deficient mice therefore supports the interpretation that METTL18 is required to maintain pituitary endocrine competency rather than upstream TRH production. Central hypothyroidism is known to blunt metabolic rate and limit somatic growth, and thus likely acts synergistically with impaired IGF‐1–mediated signaling to restrict postnatal body size in *Mettl18* KO mice. Additionally, we found that the *Ttr* gene was significantly downregulated in *Mettl18* KO embryos (Figure 3C and Table S2). It is known that transthyretin (TTR), a transporter protein, is primarily synthesized in the liver and the cerebral cortex, and is known to transport T4 to the cerebrospinal fluid and brain [33]. Therefore, these results indicate that *Mettl18* deficiency selectively suppresses the pituitary–thyroid (TSH–T4) arm of the endocrine axis while leaving hypothalamic TRH expression intact, leading to a modest thyroid hormone–deficient state that may contribute to impaired postnatal growth.

A recent study using *Mettl18*‐deficient mice reported that METTL18‐mediated histidine methylation of the ribosomal protein RPL3 is a critical post‐translational modification that ensures translational accuracy and proteostasis in the pancreas [34]. On the other hand, the decrease in liver *Nτ*‐methylhistidine levels of Mettl18 KO mice was relatively modest (Figure 4J). This observation potentially indicates functional adaptation or redundancy within the histidine methylation pathway. Specifically, other histidine methyltransferases, such as SETD3, which is known to methylate actin [35], might compensate for the loss of METTL18. Further investigation using tissue‐specific or multi‐gene knockout models will be required to clarify the precise cooperative roles of these enzymes.

Our results indicated that Mettl18 deficiency alters iron and lipid metabolism. Mechanistically, these phenotypes are likely orchestrated through the disruption of the METTL18–RPL3 translation elongation axis. We also confirmed a significant reduction in *N*τ‐methylation levels specifically within the 60S ribosomal subunit fraction isolated from Mettl18‐deficient mouse livers (Figure S5). Since METTL18 selectively methylates a specific histidine residue on RPL3 to optimize codon reading speed during translation elongation [10], its absence presumably induces ribosomal stalling and translational stress. Emerging evidence underscores that ribosomal stress triggered by RPL3 dysregulation cross‐talks with the Transferrin/Transferrin Receptor mediated iron‐uptake machinery. This perturbation is thought to alter downstream iron‐regulatory proteins, subsequently remodeling TFRC expression, resulting in the intracellular iron overload and hypersensitivity to lipid peroxidation observed in our model. Furthermore, the METTL18–RPL3 axis appears to dictate mitochondrial energy programming. It has been reported that ribosomes harboring RPL3, but not its paralog RPL3L, physically interact with the mitochondrial outer membrane to upregulate oxidative phosphorylation and ATP production [36]. Consequently, the loss of METTL18‐mediated RPL3 methylation may disrupt this specialized ribosome–mitochondria interaction, rewiring mitochondrial lipid and energy combustion networks.

Taken together, our findings indicate METTL18 contributes to organismal growth through its effects on fetal iron handling, postnatal metabolic homeostasis, and endocrine function. This integrative view is consistent with the organism‐wide phenotypes observed across developmental, metabolic, and endocrine dimensions. During embryogenesis, METTL18 ensures adequate iron handling and fetal tissue expansion. After birth, METTL18 maintains metabolic and endocrine environments conducive to somatic growth by supporting lipid availability, IGF‐1‐mediated signaling, and pituitary–thyroid axis function. Such converging developmental, metabolic, and endocrine phenotypes point to a unified growth‐control framework supported by METTL18. Loss of METTL18 is associated with coordinated limitations—prenatal hypoplasia, metabolic constraints, and suppression of anabolic endocrine pathways—that converge to restrict postnatal somatic growth.

Although identifying the direct substrates of METTL18 in mammalian tissues remains an important future issue, our findings suggest that a single universal substrate is unlikely to account for the broad and tissue phenotypes observed in *Mettl18* KO mice. METTL18 is expressed in multiple organs—including liver, adipose tissue, pituitary, and brain—and histidine methylation has been implicated in diverse aspects of protein function and cellular regulation. It is therefore plausible that METTL18 acts on distinct sets of substrates in different tissues, or modulates the expression or activity of specific classes of mRNAs in a context‐dependent manner, rather than acting through a single molecular target. This multi‐layered regulatory model is more consistent with the systemic defects we observe, spanning prenatal development, metabolic homeostasis, and endocrine signaling. Defining the tissue‐specific and function‐specific substrates of METTL18 will require integrative biochemical, proteomic, and ribosome‐profiling approaches, and will be essential for elucidating how histidine methylation integrates biological control with systemic physiology in mammals.

## Author Contributions

F.K., J.‐D.K., and A.F. were involved in the study design and conceptualisation. F.K., K.K., N.M., Y.Y., K.M., T.S., H.M., N.K., K.N., H.D., K.H., S.N., M.Y., Y.I., and J.‐D.K. were involved in the animal and laboratory experiments. F.K., J.‐D.K., and A.F. wrote the manuscript. All authors approved the final version of the manuscript.

## Funding

This work was supported by Japan Agency for Medical Research and Development (AMED) (JPgm1410010) and MEXT | Japan Society for the Promotion of Science (JSPS) (23H00321 and 26H02313).

## Conflicts of Interest

The authors declare no conflicts of interest.

## Supporting information


**Figure S1:** Validation of LC–MS/MS‐based TRH detection. The synthetic TRH standard was determined as *m/z* 363.0 > 249.2 (upper panel). Chromatograms show that the TRH peak was readily detected in brain extracts of wild‐type mice with a retention time identical to that of the synthetic TRH standard (middle panel), whereas no corresponding peak was observed in prepro‐TRH knockout mice (lower panel).
**Figure S2:** Amino acid sequence alignment, and tissue expression of Mettl18 mRNA. (A) Sequence alignment of human and mouse METTL18 proteins underlined conserved methyltransferase motifs (motif I and II), including the invariant glycine residue mutated in this study. (B, C) Tissue distribution of Mettl18 mRNA in adult wild‐type male (B) and female (C) mice determined by RT‐PCR.
**Figure S3:** Postnatal growth and organismal growth of Mettl18 KO. (A–C) Body weight of wild‐type (Mettl18 +/+, WT), heterozygous (Mettl18+/−, Hetero), and homozygous (Mettl18−/−, KO) Male (A) and female (B) mice at P0. Representative images at P10 (C). (D) Wet weights of multiple organs (brain, lung, heart, liver, kidney, eWAT, iWAT, BAT, skeletal muscle, spleen) at 2‐month‐old WT and Mettl18 KO mice. Data are presented as mean ± standard errors of the mean for *n* = 4–18 per group. (E) Representative images showing livers (upper image) and eWAT (lower image) of 2‐month‐old WT and Mettl18 KO mice. (F) Expression analysis of genes involved in adipogenesis (Pparγ, Cebpα/β), lipolysis (Hsl, Atgl, Mgl, Plin1, Abhd5), and lipogenesis (Srebf1a/c, Scd1, Fasn, Elovl6) (*n* = 4–5). *p < 0.05, *p* < 0.01, ***p < 0.001 versus WT control. Statistical analyses were performed using unpaired two‐tailed Student's *t*‐tests.
**Figure S4:** Effects of METTL18 on blood glucose and GH levels. (A) Fed and fasted blood glucose. Data are presented as mean ± standard errors of the mean for *n* = 4 per group. (B) Plasma GH levels in fed and fasted conditions. Data are presented as mean ± standard errors of the mean for *n* = 4 per group. ***p < 0.001, ****p < 0.0001, and ns (not significant). Statistical analysis was performed using one‐way analysis of variance (ANOVA), followed by Tukey's post hoc test for multiple comparisons.
**Figure S5:** Levels of Nτ‐methylation within the 60S ribosomal subunit fraction isolated from the liver of Mettl18‐deficient mouse. (A) Sucrose density gradient for ribosomal complexes. Lysates were prepared with a buffer containing EDTA to dissociate 80S into 40S and 60S. The 60S fraction used for liquid chromatography mass spectrometry (LC–MS/MS) analysis. (B) Relative ratio of the median 40S to 60S protein intensity as quantified by mass spectrometry in the indicated sucrose gradient fractions. (C) Quantification of liver Nτ‐methylhistidine levels corresponding to fractions from the sucrose gradient in panel A. (D) Factions were resolved using 10% SDS‐PAGE followed by reverse staining. Bands 1, 2, and 3 between 40 and 60 kDa including RPL3 (46 kDa) were indicated with arrows. (E) Quantification of the amount of Nτ‐methylhistidine in the liver corresponding to bands 1, 2, and 3. Data are presented as mean ± standard errors of the mean for *n* = 3 per group. ***p < 0.001 and ****p < 0.0001. Statistical analyses were performed using unpaired two‐tailed Student's *t*‐tests.
**Table S1:** The primer sequences used in qPCR.

**Table S2:** List of all differentially expressed genes (DEGs) per comparison (Excel file).

## Data Availability

All data required to evaluate the conclusions of the paper are presented in this study or upon reasonable request from the corresponding authors. All raw data files for the RNA‐seq analysis were deposited in the NCBI for Biotechnology Information Gene Expression Omnibus (GEO) database (https://www.ncbi.nlm.nih.gov/geo; accession number: GSE314501).
