# Supplementary material for: METTL18 is Required for Developmental, Metabolic, and Endocrine Regulation of Mammalian Growth
Source: FASEB J. 2026 Aug 27;40(17):e72224. doi: 10.1096/fj.202600392R (PMC13521299; doi:10.1096/fj.202600392R)
Supplement: Supplementary file 1 [file FSB2-40-e72224-s001.pdf]

# Supplementary Information

## METTL18 is required for developmental, metabolic, and endocrine regulation of mammalian growth

Fumiya Kasai, Jun-Dal Kim[#], Koichiro Kako, Naoto Muromachi, Takeru Saito, Yao Yuan, Hayase Mizukami, Nana Kishikawa, Kanako Nakamura, Kaori Motomura, Hiroaki Daitoku, Yuuki Imai, Sho Sekiguchi, Kazuhiko Horiguchi, Satoshi Narumi, Masanobu Yamada, and Akiyoshi Fukamizu[#]

[#]**Co-correspondence**: Jun-Dal Kim (jdkim@inm.u-toyama.ac.jp);
Akiyoshi Fukamizu (akif@tara.tsukuba.ac.jp)

**Supplementary Figures S1-S5**

**Supplementary Tables S1 and S2 (Excel file)**

# Supplementary Figure S1

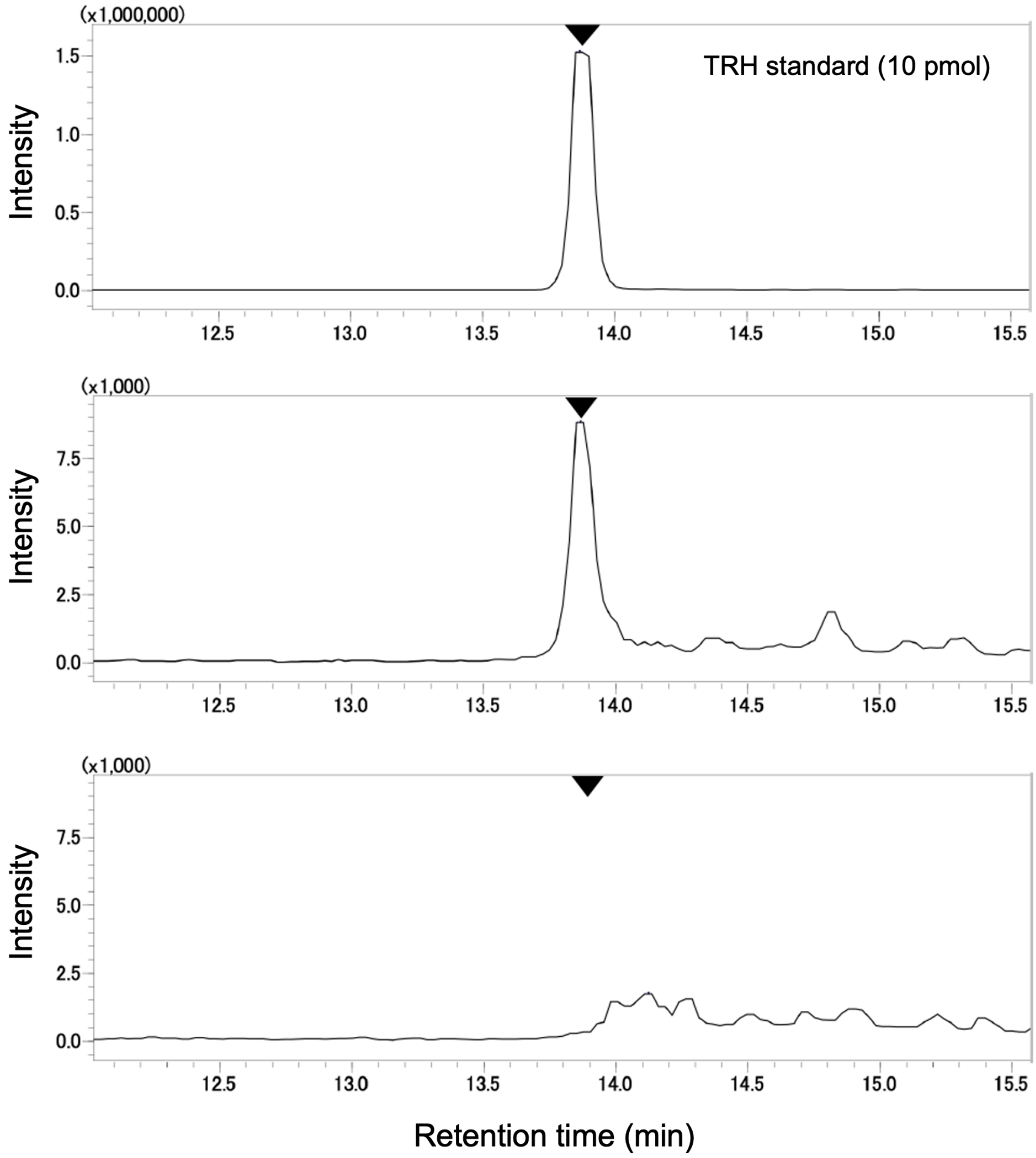


**Supplementary Figure S1. Validation of LC-MS/MS-based TRH detection.** The synthetic TRH standard was determined as *m/z* 363.0 > 249.2 (upper panel). Chromatograms show that the TRH peak was readily detected in brain extracts of wild-type mice with a retention time identical to that of the synthetic TRH standard (middle panel), whereas no corresponding peak was observed in prepro-TRH knockout mice (lower panel).

# Supplementary Figure S2

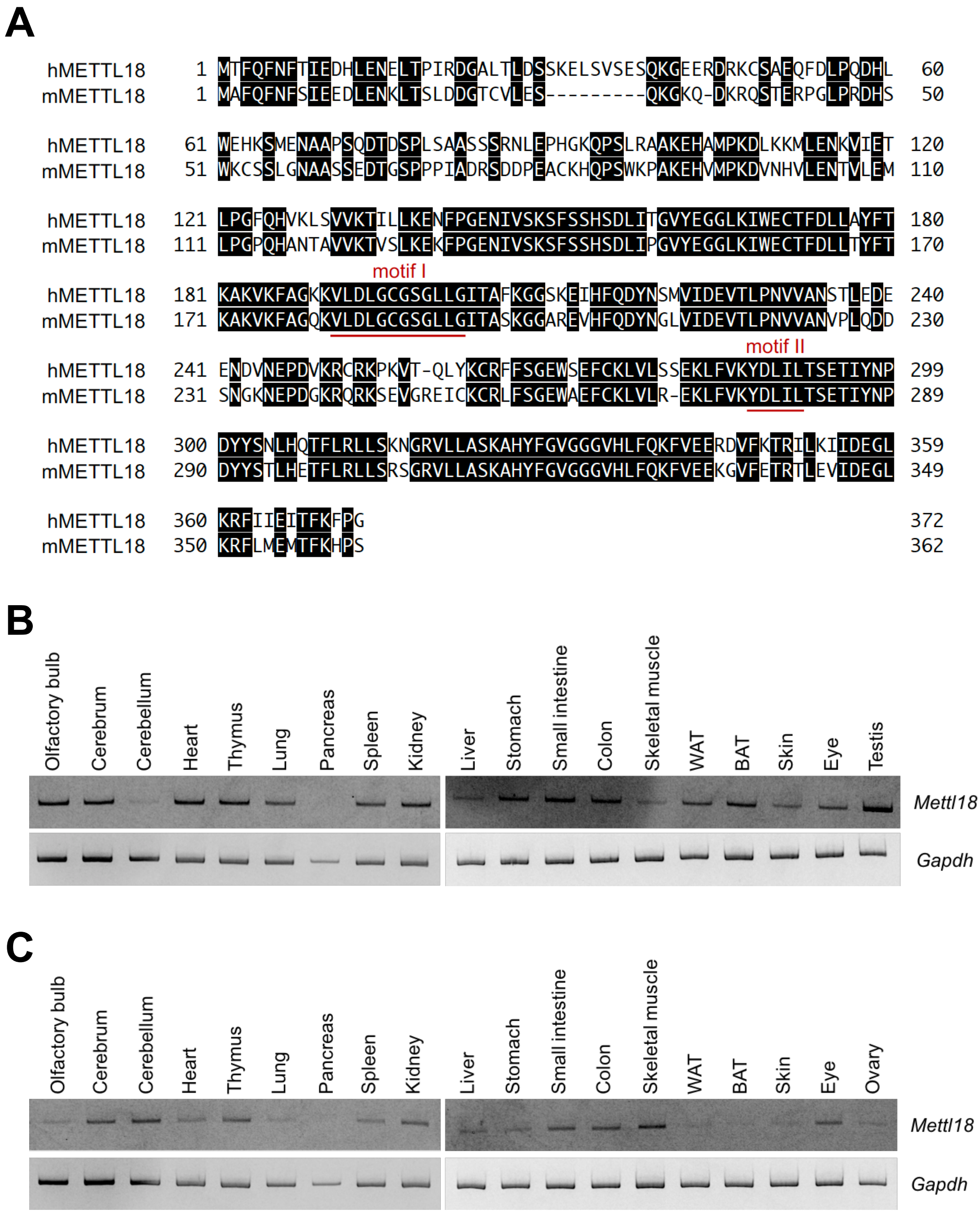


**Supplementary Figure S2. Amino acid sequence alignment, and tissue expression of *Mettl18* mRNA.** (**A**) Sequence alignment of human and mouse METTL18 proteins underlined conserved methyltransferase motifs (motif I and II), including the invariant glycine residue mutated in this study. (**B, C**) Tissue distribution of *Mettl18* mRNA in adult wild-type male (B) and female (C) mice determined by RT–PCR.

## Supplementary Figure S3

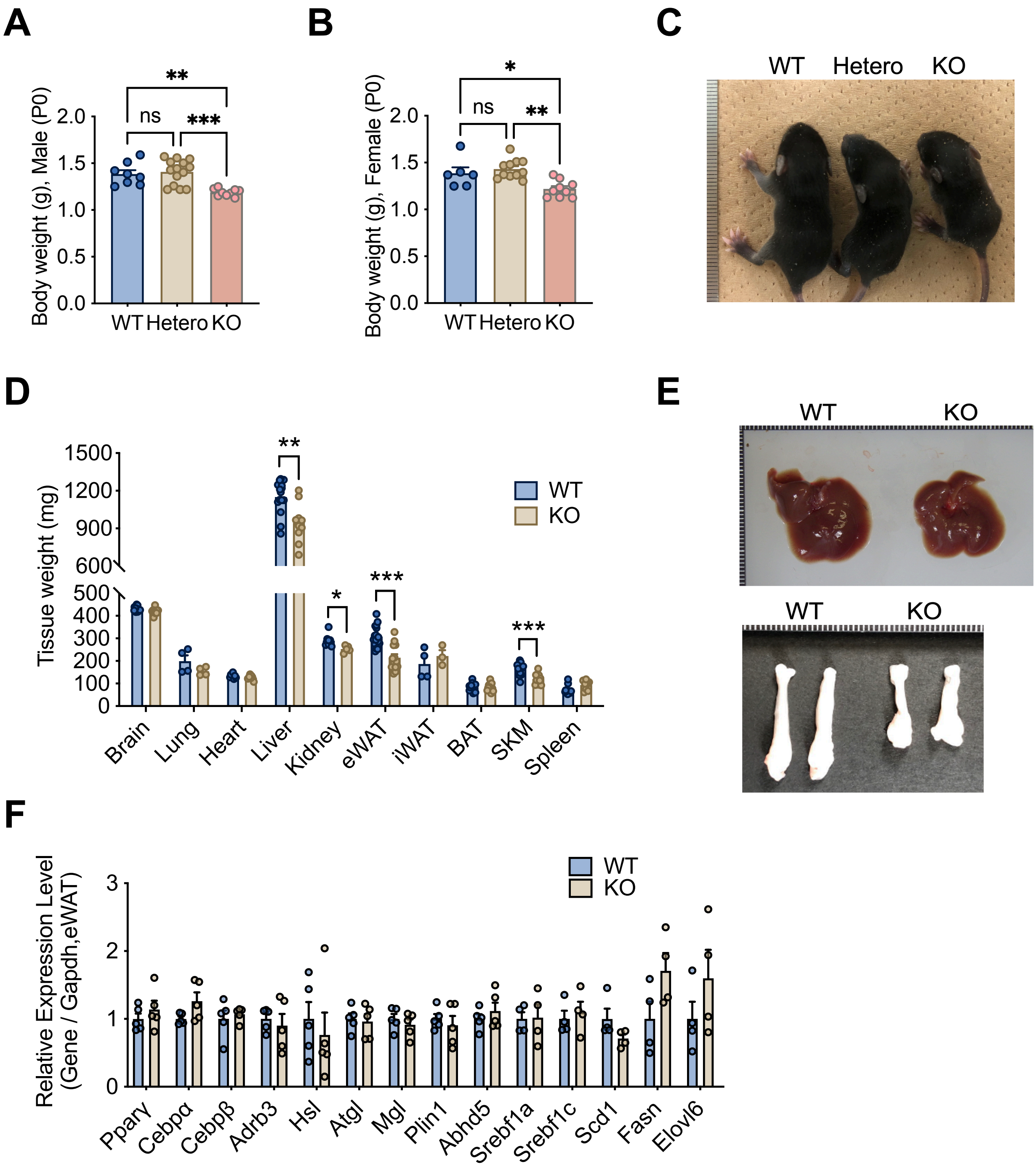


**Supplementary Figure S3. Postnatal growth and organismal growth of *Mettl18* KO.** (**A-C**) Body weight of wild-type (*Mettl18* +/+, WT), heterozygous (*Mettl18*+/–, Hetero), and homozygous (*Mettl18*–/–, KO) Male (A) and female (B) mice at P0. Representative images at P10 (C). (**D**) Wet weights of multiple organs (brain, lung, heart, liver, kidney, eWAT, iWAT, BAT, skeletal muscle, spleen) at 2-month-old WT and *Mettl18* KO mice. Data are presented as mean ± standard errors of the mean for n = 4-18 per group. (**E**) Representative images showing livers (upper image) and eWAT (lower image) of 2-month-old WT and *Mettl18* KO mice. (**F**) Expression analysis of genes involved in adipogenesis (*Pparγ, Cebpα/β*), lipolysis (*Hsl, Atgl, Mgl, Plin1, Abhd5*), and lipogenesis (*Srebf1a/c, Scd1, Fasn, Elovl6*) (n=4-5). **p* < 0.05, ***p* < 0.01, ****p* < 0.001 vs. WT control. Statistical analyses were performed using unpaired two-tailed Student's t-tests.

## Supplementary Figure S4

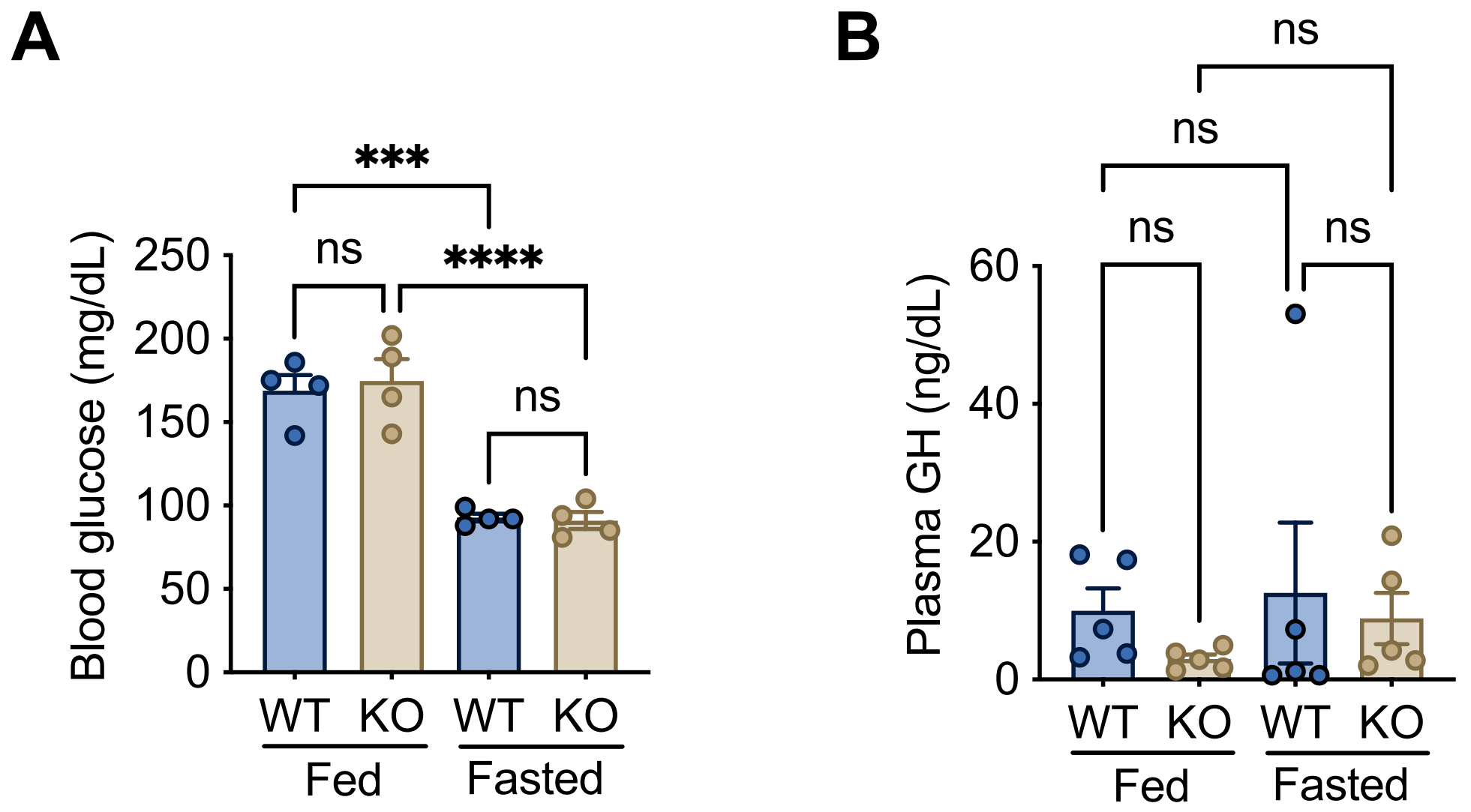


**Supplementary Figure S4. Effects of METTL18 on blood glucose and GH levels. (A)** Fed and fasted blood glucose. Data are presented as mean ± standard errors of the mean for n = 4 per group. (**B**) Plasma GH levels in fed and fasted conditions. Data are presented as mean ± standard errors of the mean for n = 4 per group. *** $p < 0.001$, **** $p < 0.0001$, and ns (not significant). Statistical analysis was performed using one-way analysis of variance (ANOVA), followed by Tukey's post-hoc test for multiple comparisons.

# Supplementary Figure S5

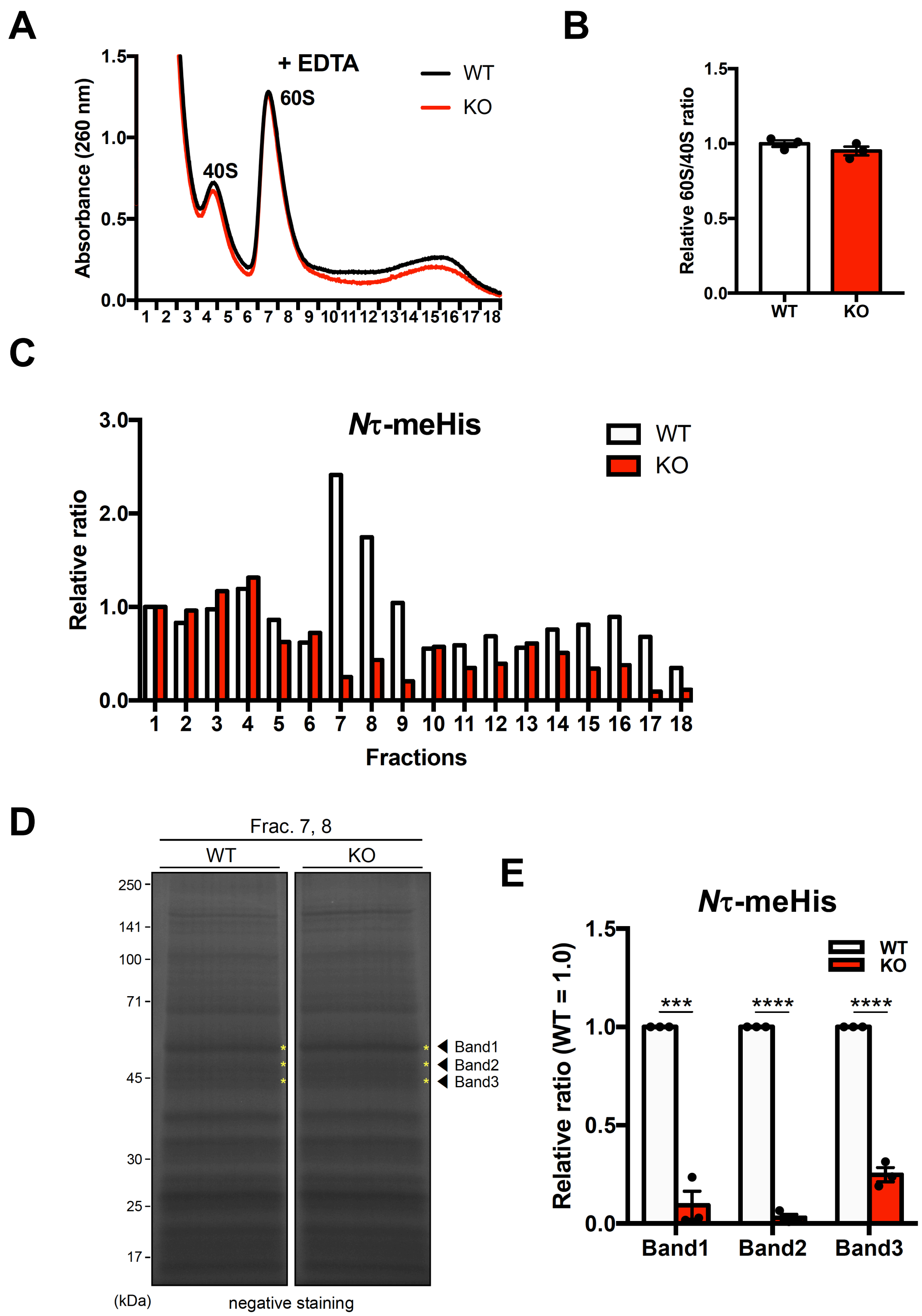


**Supplementary Figure S5. Levels of *N*τ-methylation within the 60S ribosomal subunit fraction isolated from the liver of Mettl18-deficient mouse. (A)** Sucrose density gradient for ribosomal complexes. Lysates were prepared with a buffer containing EDTA to dissociate 80S into 40S and 60S. The 60S fraction used for liquid chromatography mass spectrometry (LC-MS/MS) analysis. (**B**) Relative ratio of the median 40S to 60S protein intensity as quantified by mass spectrometry in the indicated sucrose gradient fractions. (**C**) Quantification of liver *N*τ-methylhistidine levels corresponding to fractions from the sucrose gradient in panel A. (**D**) Factions were resolved using 10% SDS-PAGE followed by reverse staining. Bands 1, 2, and 3 between 40 and 60 kDa including RPL3 (46 kDa) were indicated with *arrows*. (**E**) Quantification of the amount of Nτ-methylhistidine in the liver corresponding to bands 1, 2, and 3. Data are presented as mean ± standard errors of the mean for n = 3 per group. *** $p < 0.001$ and **** $p < 0.0001$. Statistical analyses were performed using unpaired two-tailed Student's t-tests.

# Supplementary Tables

## Supplementary Table S1. The primer sequences used in qPCR.

| Gene | Forward | Reverse |
|---|---|---|
| *Mettl18* | CAAGATGACAGCAATGGCAAA | CCCACTCCCCAGAGAACAAG |
| *Trf* | TGCATCAAGGCCATTTCTGC | CCGGCATCGTACACCCAAC |
| *Tfrc* | TGCTATAGGTCCTGAGGGCAT | GGCATACAGCTCAATGGAAGA |
| *Pparg* | CTTGTGAAGGATGCAAGGGTT | AGGCACTTCTGAAACCGACA |
| *Cebpa* | GCAAAGCCAAGAAGTCGGTG | TCACTGGTCAACTCCAGCAC |
| *Cebpb* | CACAAGGTGCTGGAGCTGA | TTGAACAAGTTCCGCAGGGT |
| *Adrb3* | AGACAGGGACAGAGGGGTTG | GTGGGAATTGGAGGGTGGAG |
| *Hsl* | CACCCATAGTCAAGAACCCCTTC | TCTACCACTTTCAGCGTCACCG |
| *Atgl* | GTGAAGCAGGTGCCAACATTATTG | AAACACGAGTCAGGGAGATGCC |
| *Mgl* | GCTGGTATTTGCCCATGACC | CCGACACCACCATCCTCTCT |
| *Plin1* | TACACTATGTGCCGCTTCCC | CGCCTCTGCTGAAGGGTTAT |
| *Abhd5* | TCTTGCTTGGACACAACCTG | GAGGTGACTAACCCTTGATGG |
| *Srebf1a* | AGGCGGCTCTGGAACAGA | TCAAAACCGCTGTGTCCAGTT |
| *Srebf1c* | CGGCGCGGAAGCTGT | TGCAATCCATGGCTCCGT |
| *Scd1* | AGATCTCCAGTTCTTACACGACC | CTTTCATTCAGGACGGATGTCT |
| *Fasn* | CAAATACAATGGCACCCTGA | TGGCGAAGCCGTAGTTAGTT |
| *Elovl6* | ACAATGGACCTGTCAGCAAA | CTACCAGTGCAGGAAGATCAGT |
| *Gapdh* | TCACTGGCATGGCCTTCC | CAGGCGGCACGTCAGATC |

## Supplementary Table S2. List of all differentially expressed genes (DEGs) per comparison (Excel file).
